# Factors Associated with Mortality in Coronavirus-Associated Mucormycosis: Results from Mycotic Infections in COVID-19 (MUNCO) Online Registry

**DOI:** 10.3390/jcm11237015

**Published:** 2022-11-27

**Authors:** Shitij Arora, Shivakumar Narayanan, Melissa Fazzari, Kranti Bhavana, Bhartendu Bharti, Shweta Walia, Neetu Kori, Sushila Kataria, Pooja Sharma, Kavya Atluri, Charuta Mandke, Vinod Gite, Neelam Redkar, Mayank Chansoria, Sumit Kumar Rawat, Rajani S. Bhat, Ameet Dravid, Yatin Sethi, Chandan Barnawal, Nirmal Kanti Sarkar, Sunit Jariwala, William Southern, Yoram Puius

**Affiliations:** 1 Division of Hospital Medicine, Montefiore Medical Center, Albert Einstein College of Medicine, NW651, 111 E 210th Street, Bronx, NY 10467, USA; 2 Institute of Human Virology, University of Maryland School of Medicine, Baltimore, MD 21201, USA; 3 Division of Biostatistics, Albert Einstein College of Medicine, Bronx, NY 10461, USA; 4 All India Institute of Medical Sciences, Patna 801507, Bihar, India; 5 Maharaja Yeshwantrao Hospital, Indore 452010, Madhya Pradesh, India; 6 Medanta Institute of Education & Research, Gurugram 122018, Haryana, India; 7 Department of Bioinformatics, University of California, Los Angeles, CA 90095, USA; 8 Department of Ophthalmology, Hinduhridaysamrat Balasaheb Thackarey Medical College and Dr. Rustom Narsi Cooper Municipal General Hospital, Mumbai 400056, Maharashtra, India; 9 Department of ENT, Hinduhridaysamrat Balasaheb Thackarey Medical College and Dr. Rustom Narsi Cooper Municipal General Hospital, Mumbai 400056, Maharashtra, India; 10 Department of Medicine, Hinduhridaysamrat Balasaheb Thackarey Medical College and Dr. Rustom Narsi Cooper Municipal General Hospital, Mumbai 400056, Maharashtra, India; 11 Department of Emergeny Medicine, Netaji Subhash Chandra Bose Medical College, Jabalpur 482003, Madhya Pradesh, India; 12 Bundelkhand Medical College Sagar, Sagar 470001, Madhya Pradesh, India; 13 Consultant Pulmonologist, Bangalore 560080, Karnataka, India; 14 Noble Hospital, Pune 411013, Maharashtra, India; 15 Venkateshwara Hospital, Dwarka, New Delhi 110075, Delhi, India; 16 Associate Consultant and Academic Co-Ordinator Nepal MediCiti, Karyabinayak 44600, Nepal; 17 Sarkari Kormochari Hospital, Dhaka 1212, Bangladesh; 18 Biodesign and Innovation Program, Montefiore Medical Center, Albert Einstein College of Medicine, New York, NY 10461, USA; 19 Division of Infectious Diseases, Montefiore Medical Center, Albert Einstein College of Medicine, New York, NY 10461, USA

**Keywords:** coronavirus, mucormycosis, steroids

## Abstract

Background: COVID-19-associated mucormycosis (CAM) is associated with high morbidity and mortality. MUNCO is an international database used to collect clinical data on cases of CAM in real time. Preliminary data from the Mycotic Infections in COVID-19 (MUNCO) online registry yielded 728 cases from May to September 2021 in four South Asian countries and the United States. A majority of the cases (694; 97.6%) consisted of a mucormycosis infection. The dataset allowed for the analysis of the risk factors for adverse outcomes from CAM and this analysis is presented in this paper. Methods: The submission of cases was aided by a direct solicitation and social media online. The primary endpoints were full recovery or death measured on day 42 of the diagnosis. All patients had histopathologically confirmed CAM. The groups were compared to determine the contribution of each patient characteristic to the outcome. Multivariable logistic regression models were used to model the probability of death after a CAM diagnosis. Results: The registry captured 694 cases of CAM. Within this, 341 could be analyzed as the study excluded patients with an unknown CAM recovery status due to either an interruption or a lack of follow up. The 341 viable cases consisted of 258 patients who survived after the completion of treatment and 83 patients who died during the period of observation. In a multivariable logistic regression model, the factors associated with an increased risk of mortality include old age (OR = 1.04, 95% CI 1.02–1.07, *p* = 0.001), history of diabetes mellitus (OR 3.5, 95% CI 1.01–11.9, *p* = 0.02) and a lower BMI (OR 0.9, 95% CI 0.82–0.98, *p* = 0.03). Mucor localized to sinus disease was associated with 77% reduced odds of death (OR = 0.23, 95% CI 0.09–0.57, *p* = 0.001), while cerebral mucor was associated with an increased odds of death (OR = 10.96, 95% CI 4.93–24.36, *p* = ≤0.0001). Conclusion: In patients with CAM, older age, a history of diabetes and a lower body mass index is associated with increased mortality. Disease limited to the sinuses without a cerebral extension is associated with a lower risk of mortality. Interestingly, the use of zinc and azithromycin were not associated with increased mortality in our study.

## 1. Introduction

As of 12 October 2022, over 619 million confirmed cases of COVID-19 and 6.5 million deaths have been globally reported [1]. The SARS-CoV-2 virus emerged in 2019, leading to the beginning of the COVID-19 pandemic. A co-infection of COVID-19 can cause a serious illness and impairment, especially in those who are immunocompromised. A significant increase in cases of mucormycosis, a relatively rare fungal infection, has been seen in patients with COVID-19, termed COVID-19-associated mucormycosis (CAM). The pathophysiology is thought to be related to COVID-19-associated pulmonary aspergillosis (CAPA) and influenza-associated pulmonary aspergillosis (IAPA). CAM emerged as a significant healthcare challenge, with more than 41,000 cases reported as of September 2021 in India alone [2]. It is a life-threatening infection and carries high morbidity and mortality. Several risk factors are associated with an increased likelihood of acquiring the disease and they are seen more commonly in patients with underlying immunosuppression corticosteroid therapy and uncontrolled diabetes, with or without diabetic ketoacidosis [3]. There are other suggested hypotheses, including the role of a zinc supplementation and iron overload states in CAM that remain untested. Zinc is a micronutrient for fungal growth and has been shown to augment growth in vitro [3,4].

Mortality, even with standard care, is high at around 45–50% for rhino-orbital disease, >90% for disseminated disease and it is higher for patients with malignancies (66%) or diabetes (45%) than for immunocompetent hosts (35%) [4]. Although India emerged as the epicenter for CAM, sporadic cases comprised of various mycotic infections aside from mucormycosis were reported from several countries across the world. We established an online registry (Mycotic Infections in COVID-19; MUNCO) to collect clinic–epidemiologic data on CAM and other fungal infections using an online reporting system in real time. Our findings from April to June 2021 were previously reported from the first 65 cases of CAM in the registry [5]. Due to the severity of CAM, urgency was placed on the data collection and analysis [5]. At the time, the number of reported cases available provided a limited ability to fully evaluate the risk factors associated with adverse outcomes in CAM. In this report, we sought to further evaluate the association of the clinic–epidemiologic factors associated with mortality in CAM.

## 2. Methods

### 2.1. Data Collection

Cases were collected from July 2021 to June 2022 via an online questionnaire submitted by international physicians to report the case and treatment of CAM in a known patient. This was collected through a REDCap database [6] at http://covidmucor.com, as previously described [5]. The cases were entered at the discretion of the reporting physician, but the case definition included a histopathologically confirmed infection. The outcomes were defined as a full recovery or death at the 6-month time point. Online solicitation for the cases was performed through social media and networking. The authors confirm that the ethical policies of the journal, as noted on the journal’s author guidelines page, have been adhered to and the appropriate ethical review committee approval has been received.

### 2.2. Statistical Analysis

Descriptive statistics such as the mean with standard deviation (SD), median with an inter-quartile range (IQR) and frequencies (*n*, %) were generated to summarize the patient characteristics both overall and stratified by the outcome (full recovery, death or a composite of death or vision loss). For each patient characteristic, the groups were formally compared via chi-square or Fisher’s exact test for the association (categorical), two-sample *t*-test or Wilcoxon test (continuous). A multivariable logistic regression model was estimated to model the probability of death after a CAM diagnosis during treatment using Firth’s penalized maximum likelihood estimates. Our primary variables of interest included a history of diabetes mellitus, obesity, the location of the disease, zinc and corticosteroid treatments which were examined along with a set of pre-selected covariates (age, location of and days to mucor infection and any known previous ICU stay) assumed to be potential confounders. Odds ratios, 95% confidence intervals and p-values corresponding to Wald chi-square tests for association were generated to summarize the effects. Two-sided *p*-values less than 0.05 were considered to be statistically significant. All analyses were conducted using SAS version 9.4 (SAS Institute Inc., Cary, NC, USA, copyright 2016).

## 3. Results

A total of 694 patients diagnosed with CAM were recorded in the database. Of these, 353 patients were still alive but had an unknown CAM recovery status due to an interrupted or failure to follow-up, and were thus excluded. Thus, a total of 341 patients were retained in the analysis data set, consisting of 258 patients who successfully completed treatment and survived versus the 83 patients who died.

Table 1a compares the baseline characteristics between survivors and non-survivors as well as the overall dataset. The patient ages ranged from 38 to 64 and the mean age of all the CAM patients was 51.7 years (S.D. 13 years). In total, 79% of the patients were female. The mean body mass index was 24.7 (S.D. 4). Of the overall population, 31% were overweight, 8% were obese and 4.5% were classified as underweight based on their BMI. Among subjects with CAM, 84% (286 patients) were diabetic and 21% (72 patients) had hypertension. The median hemoglobin A1C was 8.8 (IQR 7.4–10.9). A total of 85% of the overall population was unvaccinated. Among the CAM patients with diabetes, 11 of them (3.2%) had a presentation with ketoacidosis at the time of their COVID-19 diagnosis. Other potential risk factors were observed infrequently, such as a history of cancer (0.3%), an organ transplant (2.1%) or an HIV infection (0.3%). The median time from a COVID-19 diagnosis to the diagnosis of CAM was 20 days (IQR 14–30). Patients with CAM had higher C-reactive protein [54.3 mg/L (IQR 22.6–98.5)] and ferritin levels [509 ng/mL (306–931)].

In total, 85.6% (292/341) of all patients were treated with corticosteroids for COVID-19. The median steroid dose was 50 mg prednisone equivalent, with 52% of the overall population receiving >10 days of steroid therapy. The missing data in our data set included vaccination status (11.7%), gender (0.6%), BMI (2.1%), days to mucormycosis infection (10.6%), CRP (49.6%), Ferritin (29.3%), A1c (31.4%), steroid dose (52.5%), steroid duration (30.2%) and steroid type (31.1%).

Overall, 83 patients died due to CAM (24.3%). The mean age of the non-survivors was 6.8 years higher than the survivors (56.9 vs. 50.1 years). The non-survivors had a lower BMI (23.87) than the survivors (25.01) and out of the 15 underweight patients, 10 were non-survivors (66.7%). The non-survivors also had a higher median A1C of 9.6 (IQR 8.3–11.8) compared to 8 (IQR 6.9–10.0) among the survivors. The median time from the COVID-19 diagnosis to the diagnosis of CAM was shorter (17 days, IQR 11–27) in non-survivors as compared to survivors (21 days, IQR 15–30, *p* = 0.007). The non-survivors also had higher levels of CRP and ferritin as compared to the survivors (median ferritin 85.1 mg/L, IQR 47–119 vs. 40.2 mg/L, IQR 18–70; median ferritin 763 ng/mL, IQR 373–1174 vs. 359.5 ng/mL, IQR 234–578, *p* < 0.001). Among those treated with corticosteroids, the non-survivors were treated with a higher median daily dose of 53 mg prednisone equivalent, (IQR 50–100) as compared to the survivors (median dose 50 mg prednisone equivalent, IQR 40–53.3), though a longer duration did not result in a higher mortality risk when looking at steroid courses ≥10 days or <10 days.

Table 1b includes the anatomical location of CAM. The most common site was sinus disease (307 patients, 90%) overall, in both the survivors and non-survivors. Out of the 307 patients with sinus disease, 239 (77.9%) were survivors while 68 (22.1%) were non-survivors. The non-survivors had a higher percentage of cerebral disease (67.3%) and pulmonary disease (75%). An orbital progression occurred in 183 patients (54%), while a cerebral extension occurred in 52 patients (15%). Rarer manifestations included pulmonary mucormycosis (12 patients, 3.5%) and cutaneous mucormycosis (4 patients, 1.2%), while 3 patients (0.9%) had gastric mucormycosis.

Table 1c shows the medications used during the course of treatment for CAM. These include amphotericin B (286 patients, 83.9%) with or without Posaconazole (202 patients, 59.2%). Isavuconazole (22 patients, 6.5%), voriconazole (6 patients, 1.8%) and surgery (258 patients, 75.7%) were also utilized as CAM treatment options. Liposomal amphotericin B was the most commonly used preparation of amphotericin (94.1%). We also noted the use of alternate formulations in combination with, or excluded, liposomal amphotericin B, including amphotericin B lipid complex (ABLC) and conventional amphotericin B deoxycholate (16.4%). Ultimately, 258 (75.7%) patients received a surgical intervention, with this number consisting of 49 non-survivors (19%).

Table 1d shows the treatments given to patients for the management of COVID-19 prior to a diagnosis of CAM. Among COVID-19 therapies, there was no association with a higher risk of CAM mortality with any individual antiviral, antibiotic use or immunomodulator therapy use. Antiviral therapies included remdesivir (161 patients, 47.2%), favipiravir (93 patients, 27.3%) and ivermectin (146 patients, 42.8%). The antibiotics prescribed included azithromycin (97 patients, 28.4%) and doxycycline (138 patients, 40.5%).

In a multivariable logistic regression model (Table 2), a lower BMI (OR 0.9, *p* = 0.03) and a history of diabetes mellitus was associated with increased mortality (OR 3.5, 95% CI 1.01–11.93, *p* = 0.02). Mucor localized to sinus disease was associated with a 77% reduced odds of death (OR = 0.23, *p* = 0.001). Neither azithromycin nor a zinc treatment were associated with the probability of death after adjustment.

## 4. Discussion

We present the risk factors for mortality from CAM from an online database of reported cases from centers predominantly located in South East Asia. Indeed, >70% of the reported cases in the literature have been from India [7]. Previous observational studies from urban centers in India have reported a 2.1-fold increase in the number of cases of mucormycosis as compared to previous years [8]. The overall mortality of cases in our registry, where data were ascertainable, was 24%. This is consistent with the other published reports [7,8,9]. The reported mortality has varied from 14% to 64% and may reflect different levels of COVID severity [10], CAM severity and the aggressiveness of a surgical intervention [11] and the time of the ascertainment of the data. Mortality was lowest (14–17%) in studies which reported a high level of surgical intervention and a low COVID-19 disease acuity [11,12]. At the 6-month follow-up, the mortality from CAM was 34% with a combined surgical and medical treatment [13].

While most studies have reported a high male preponderance in cases of CAM [7,8,11], the high female preponderance in this registry is unexplained and may reflect a reporting bias. Consistent with previous studies, our data shows a high proportion of patients with diabetes, and this has a significant association with a reduced survival. The increased risk of mucormycosis in diabetes is well described in earlier studies [8,9] in patients with or without COVID-19 [14,15]. While the exact mechanism is unknown, it is suggested that due to changes in the iron metabolism, pH and the diminished phagocytic response to fungi due to hyperglycemia, as well as increased endothelial receptor expression for fungal ligands, may facilitate angioinvasion [16]. There is a bidirectional relationship between COVID-19 and diabetes where SARS-CoV2 infection may facilitate a dysglycemic state and diabetes may increase the risk of the COVID-19 infection’s severity [7,16]. India has the second-largest number of adults aged 20–79 years with diabetes [17].

Interestingly, we did find that a lower BMI was associated with increased mortality. This is not entirely surprising since ketosis and the presence of beta-hydroxybutyrate likely worsen during COVID-19 associated malnutrition states and are known to increase the expression of the host and fungal receptors that contribute to an increased tissue invasiveness [18].

Higher levels of CRP and ferritin were found in non-survivors as compared to survivors. An elevated CRP may reflect elevated IL-6 levels, and both of these can perpetuate the inflammatory diabetogenic process of COVID-19 as well [19]. Since the time to a CAM diagnosis was shorter in non-survivors as compared to survivors, they could plausibly have still been in the heightened inflammatory phase, which may predispose to an invasive and severe disease.

Corticosteroids are known risk factors for mucormycosis. COVID-19 was treated with corticosteroids in 76–87% of the published data on CAM [8,11] and in the study by Patel et al., a corticosteroid dose was appropriate in only a third of treated COVID-19 patients [8]. In our analysis, neither a corticosteroid treatment dose nor its duration was associated with a decreased survival. Prior studies hypothesized that a zinc supplementation would increase the risk of mucormycosis as it may act as a micronutrient for fungal growth [20,21]. Interestingly, in our analysis, neither a zinc treatment nor azithromycin were associated with increased mortality.

Similar to the published data in non-CAM settings [14,22,23], a disease limited to the sinus and surgical debridement are associated with lower mortality, while a CNS extension was associated with a higher mortality risk. This was consistent with our analysis that cerebral disease had much higher odds for death. A surgical intervention has been shown to reduce mortality in CAM patients without CNS involvement, but also reduced the progression of disease in those with CNS involvement, though these data were limited in terms of the duration of the follow-up [11]. It should be noted that the CNS extension of a disease has been noted in 21–50% of case series of CAM [11,13,24,25], while in non-CAM, the CNS extension has been described in 21–25% of the published cases in reports where this is clearly delineated [14,26].

The strength of our study is the large multinational sample size providing real world data about the risks of mortality of CAM. The simple online interface allowed for a rapid deployment and implementation. Limitations include a potential selection bias due to diverse locations with variable access to specialty care in the setting of pandemic shortages, a lack of comparable registry data for an external validation, a lack of a separate verification for the integrity of data at the entry point and a lack of complete follow-up information. Nevertheless, with a lack of widely available data about the clinical course and risks of the adverse outcomes, we feel this is important to leverage such a registry acquisition to disseminate the knowledge of an emerging disease.

## 5. Conclusions

CAM emerged as a serious life-threatening infection in the middle of the pandemic and this registry was deployed in order to generate data sets that can answer some of the key clinic–epidemiologic questions in our understanding of this disease. In this paper, we discuss the factors associated with mortality in CAM and identify the sites of the disease, old age, a low BMI and a history of diabetes as factors associated with increased mortality. We also identify that the use of zinc is not associated with increased mortality in CAM.

## Figures and Tables

**Table 1:** Baseline Characteristics.

(**a**) Baseline variables between group with recovery and deceased				
**Baseline Characteristic**	**Overall** **N = 341**	**Recovery** **N = 258**	**Death** **N = 83**	***p*** **-Value ^#^**
Age in years	51.72 (13.02)	50.07 (12.70)	56.88 (12.72)	<0.001
Vaccinated	46 (15.3%)	34 (73.9%)	12 (26.1%)	0.71
Female	269 (79.4%)	204 (75.8%)	65 (24.2%)	0.79
Male	70 (20.6%)	52 (74.3%)	18 (25.7%)	
BMI kg/m^2^	24.76 (4.11)	25.04 (4.19)	23.87 (3.73)	0.03
**BMI Category:**				
Underweight (<18.8)	15 (4.5%)	5 (33.3%)	10 (66.7%)	0.001
Normal (18.5 ≤ BMI < 25)	187 (56%)	144 (77%)	43 (23%)	
Overweight (25 ≤ BMI < 30)	105 (31.4%)	83 (79%)	22 (21%)	
Obese (≥30)	27 (8.1%)	22 (81.5%)	5 (18.5%)	
**Comorbidities:**				
Hypertension	72 (21.1%)	50 (69.4%)	22 (30.6%)	0.17
DM	286 (83.9%)	208 (72.7%)	78 (27.3%)	0.004
DM with ketoacidosis	11 (3.2%)	3 (27.3%)	8 (72.7%)	0.001
Cancer	1 (0.3%)	0 (0%)	1 (100%)	0.08
Organ Transplant	7 (2.1%)	5 (71.4%)	2 (28.6%)	0.79
IDU	4 (1.2%)	1 (25%)	3 (75%)	0.05
HIV+	1 (0.3%)	1 (100%)	0 (0%)	0.57
Asthma	3 (0.9%)	2 (66.7%)	1 (33.3%)	0.72
**Laboratory values:**				
CRP mg/L	54.3 (22.6–98.5)	40.2 (18.0–69.6)	85.1 (47.0–118.7)	<0.001
Ferritin ug/L	509 (306–931)	359.5 (234–578)	763 (372.9–1174)	<0.001
A1c%	8.8 (7.4–10.9)	8.0 (6.9–10.0)	9.6 (8.3–11.8)	<0.001
**Days from COVID-19 diagnosis to mucor**	20 (14–30)	21 (15–30)	17 (11–27)	0.01
**Corticosteroid Treatment**	292 (85.6%)	219 (75%)	73 (25%)	0.49
Dose, prednisone equivalent	50 (40–53.3)	50 (40–53.3)	53.3 (50–100)	<0.001
Type: Dexamethasone	132 (56.2%)	101 (76.5%)	31 (23.5%)	0.43
Methylrednisone	81 (34.5%)	56 (69.1%)	25 (30.9%)	
Prednisone	22 (9.4%)	15 (68.2%)	7 (31.8%)	
Treatment duration 10+ days	124 (52.1%)	98 (79%)	26 (21%)	0.03
(**b**) Mucor location/severity between CAM recovered and deceased				
**Location(s) of Mucor Infection**	**Overall** **N = 341 (100%)**	**Recovery** **N = 258**	**Death** **N = 83**	***p*** **-Value ^#^**
Sinus	307 (90%)	239 (77.9%)	68 (22.1%)	0.005
Pulmonary	12 (3.5%)	3 (25%)	9 (75%)	0.001
Cutaneous	4 (1.2%)	2 (50%)	2 (50%)	0.229
Gastric	3 (0.9%)	3 (100%)	0 (0%)	0.324
Ophthalmic	183 (53.7%)	133 (72.7%)	50 (27.3%)	0.167
Cerebral	52 (15.2%)	17 (32.7%)	35 (67.3%)	0.001
(**c**) Medications administered to patients during course of treatment for CAM				
**Mucor Treatment(s)**	**Overall** **N = 341 (100%)**	**Recovery** **N = 258**	**Death** **N = 83**	***p*** **-Value ^#^**
Amphotericin B	286 (83.9%)	217 (75.9%)	69 (24.1%)	0.833
Posaconazole	202 (59.2%)	164 (81.2%)	38 (18.8%)	0.004
Isavuconazole	22 (6.5%)	18 (81.8%)	4 (18.2%)	0.486
Surgery	258 (75.7%)	209 (81%)	49 (19%)	0.001
Voriconazole	6 (1.8%)	5 (83.3%)	1 (16.7%)	0.659
Amphotericin B regimen(s)				
Amphotericin B deoxycholate	47 (16.4%)	37 (78.7%)	10 (21.3%)	0.617
Liposomal amphotericin B	269 (94.1%)	209 (77.7%)	60 (22.3%)	0.004
Amphotericin B lipid complex, ABLC	39 (13.6%)	27 (69.2%)	12 (30.8%)	0.297
Amphotericin B cholesteryl sulfate complex	0 (0%)	0 (0%)	0 (0%)	0.297
(**d**) Medications administered to patients during course of treatment for COVID-19				
**COVID-19 Treatment(s)**	**Overall** **N = 341 (100%)**	**Recovery** **N = 258**	**Death** **N = 83**	***p*** **-Value ^#^**
Favipiravir	93 (27.3%)	76 (81.7%)	17 (18.3%)	0.110
Remdesivir	161 (47.2%)	121 (75.2%)	40 (24.8%)	0.837
Doxycycline	138 (40.5%)	105 (76.1%)	33 (23.9%)	0.880
Azithromycin	97 (28.4%)	74 (76.3%)	23 (23.7%)	0.865
Ivermectin	146 (42.8%)	118 (80.8%)	28 (19.2%)	0.055
Tocilizumab	14 (4.1%)	8 (57.1%)	6 (42.9%)	0.099
Itolizumab	1 (0.3%)	1 (100%)	0 (0%)	0.570
Zinc	216 (63.3%)	166 (76.9%)	50 (23.1%)	0.500
Other	35 (10.3%)	20 (57.1%)	15 (42.9%)	0.007

Data are presented as N (%), mean (SD) or median (25th–75th percentile). ^#^ Corresponds to a chi-square or Fisher’s exact test for association (categorical), two-sample *t*-test (mean (SD) presented) or Wilcoxon test (if median (IQR) presented. BMI: body mass index; DM: diabetes mellitus; IDU: injecting drug user; HIV: human immunodeficiency virus; CRP: C-reactive protein; IQR: interquartile range.

**Table 2:** Logistic regression model for the probability of death.

	Estimated Odds Ratio	*p*-Value
Patient age, years	1.04 (1.02, 1.07)	0.001
Azithromycin treatment	0.99 (0.49, 2.03)	0.76
Zinc treatment	0.76 (0.37, 1.57)	0.46
History of DM	3.47 (1.01, 11.93)	0.02
BMI, kg/m^2^	0.90 (0.82, 0.98)	0.03
Steroid treatment *Ref: no steroid treatment*	1.67 (0.68, 4.12)	0.22
Known ICU stay *Ref: no known ICU stay*	1.50 (0.70, 3.25)	0.16
Days to mucor *(continuous)*	0.98 (0.96, 1.00)	0.15
Location of mucor: Sinus *Ref: not sinus*	0.23 (0.09, 0.57)	0.001
Ophthalmic *Ref: not ophthalmic*	0.87 (0.45, 1.69)	0.61
Cerebral *Ref: not cerebral*	10.96 (4.93, 24.36)	<0.0001

DM: diabetes mellitus; BMI: body mass index; ICU: intensive care unit.

## Data Availability

The data presented in this study are available on request from the corresponding author. The data are not publicly available due to redcap policies.
